# 4-Chloro-2-nitro­benzoic acid–pyrazine (2/1)

**DOI:** 10.1107/S1600536811046113

**Published:** 2011-11-09

**Authors:** Kazuma Gotoh, Hiroyuki Ishida

**Affiliations:** a Department of Chemistry, Faculty of Science, Okayama University, Okayama 700-8530, Japan

## Abstract

In the title co-crystal, 2C_7_H_4_ClNO_4_·C_4_H_4_N_2_, the pyrazine mol­ecule is located on an inversion centre, so that the asymmetric unit consists of one mol­ecule of 4-chloro-2-nitro­benzoic acid and a half-mol­ecule of pyrazine. The components are connected by O—H⋯N and C—H⋯O hydrogen bonds, forming a 2:1 unit. In the hydrogen-bonded unit, the dihedral angle between the pyrazine ring and the benzene ring of the benzoic acid is 16.55 (4)°. The units are linked by inter­molecular C—H⋯O hydrogen bonds, forming a sheet structure parallel to (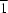
04). A C—H⋯O hydrogen-bond linkage is also observed between these sheets.

## Related literature

For related structures, see: Gotoh & Ishida (2009 ▶); Gotoh *et al.* (2010 ▶); Ishida *et al.* (2001 ▶).
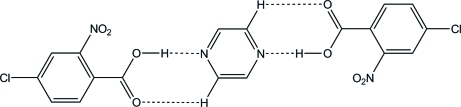


## Experimental

### 

#### Crystal data

2C_7_H_4_ClNO_4_·C_4_H_4_N_2_
*M* *_r_* = 483.22
Monoclinic, 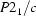

*a* = 4.87662 (13) Å
*b* = 13.5385 (3) Å
*c* = 14.7981 (6) Å
β = 90.858 (2)°
*V* = 976.89 (5) Å^3^
*Z* = 2
Mo *K*α radiation
μ = 0.39 mm^−1^
*T* = 110 K
0.35 × 0.15 × 0.11 mm

#### Data collection

Rigaku R-AXIS RAPID II diffractometer
Absorption correction: numerical (*NUMABS*; Higashi, 1999 ▶) *T*_min_ = 0.904, *T*_max_ = 0.958
19734 measured reflections
2833 independent reflections
2535 reflections with *I* > 2σ(*I*)
*R*_int_ = 0.032

#### Refinement

*R*[*F* ^2^ > 2σ(*F* ^2^)] = 0.029
*wR*(*F* ^2^) = 0.081
*S* = 1.07
2833 reflections
149 parameters
H atoms treated by a mixture of independent and constrained refinement
Δρ_max_ = 0.51 e Å^−3^
Δρ_min_ = −0.39 e Å^−3^

### 

Data collection: *PROCESS-AUTO* (Rigaku/MSC, 2004 ▶); cell refinement: *PROCESS-AUTO*; data reduction: *CrystalStructure* (Rigaku/MSC, 2004 ▶); program(s) used to solve structure: *SHELXS97* (Sheldrick, 2008 ▶); program(s) used to refine structure: *SHELXL97* (Sheldrick, 2008 ▶); molecular graphics: *ORTEP-3* (Farrugia, 1997 ▶); software used to prepare material for publication: *CrystalStructure* (Rigaku/MSC, 2004 ▶) and *PLATON* (Spek, 2009 ▶).

## Supplementary Material

Crystal structure: contains datablock(s) global, I. DOI: 10.1107/S1600536811046113/fj2468sup1.cif

Structure factors: contains datablock(s) I. DOI: 10.1107/S1600536811046113/fj2468Isup2.hkl

Supplementary material file. DOI: 10.1107/S1600536811046113/fj2468Isup3.cml

Additional supplementary materials:  crystallographic information; 3D view; checkCIF report

## Figures and Tables

**Table 1:** Hydrogen-bond geometry (Å, °)

*D*—H⋯*A*	*D*—H	H⋯*A*	*D*⋯*A*	*D*—H⋯*A*
O2—H2⋯N2	0.88 (2)	1.80 (2)	2.6739 (10)	178 (2)
C3—H3⋯O1^i^	0.95	2.51	3.4305 (12)	162
C6—H6⋯O3^ii^	0.95	2.59	3.4865 (13)	157
C8—H8⋯O1	0.95	2.55	3.2201 (12)	128
C9—H9⋯O3^iii^	0.95	2.45	3.1273 (12)	128

Symmetry codes: (i) 
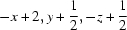
; (ii) 
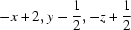
; (iii) 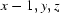.

**Fig. 1.**
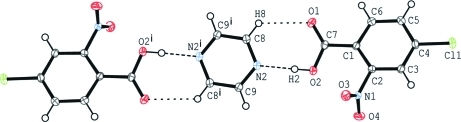
The molecular structure of the title compound, with the atom-labeling. Displacement ellipsoids of non-H atoms are drawn at the 50% probability level. The dashed lines indicate the O—H···N and C—H···O hydrogen bonds. [Symmetry code: (i) -x, -y + 1, -z.]

**Fig. 2.**
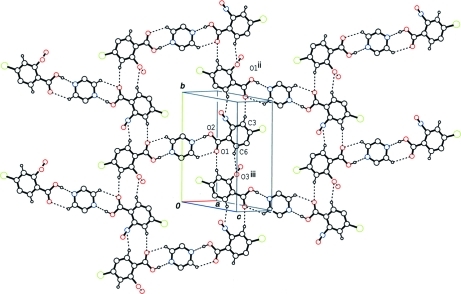
A packing diagram of the title compound, showing a sheet structure formed by O—H···N and C—H···O hydrogen bonds (dashed lines). [Symmetry codes: (ii) -x + 2, y + 1/2, -z + 1/2; (iii) -x + 2, y - 1/2, -z + 1/2.]

## supplementary crystallographic information

### Comment

The title compound was prepared in order to extend our study on *D*—H···*A* hydrogen bonding (*D* = N, O, or C; *A* = N, O or Cl) in pyridine–substituted benzoic acid systems (Gotoh & Ishida, 2009; Gotoh *et al.*, 2010). The structures of the (1/2) compounds of pyrazine with 2-chloro-4-nitrobenzoic acid and 2-chloro-5-nitrobenzoic acid have been reported (Ishida *et al.*, 2001).

In the crystal structure of the title compound, no acid-base interaction involving proton transfer is observed between the two components, which are linked by O—H···N and C—H···O hydrogen bonds (Table 1 and Fig. 1). In the hydrogen-bonded 1:2 unit located on an inversion centre, the dihedral angle between the pyrazine ring and the benzene ring of the benzoic acid is 16.55 (4)°. The carboxyl plane makes dihedral angles of 7.15 (11) and 22.01 (11)°, respectively, with the pyrazine and benzene rings. The dihedral angle between the nitro group and the benzene ring is 77.58 (11)°. The 1:2 units are linked by intermolecular C—H···O hydrogen bonds between the acid molecules (C3—H3···O1^ii^ and C6—H6···O3^iii^; Table 1), forming a sheet parallel to the (104) plane (Fig. 2). The sheets are further linked by a C—H···O hydrogen bond (C9—H9···O^iv^; Table 1), forming a three-dimensional hydrogen-bonded network.

### Experimental

Single crystals were obtained by slow evaporation from an acetonitrile solution (50 ml) of 4-chloro-2-nitrobenzoic acid (0.620 g) and pyrazine (0.123 g) at room temperature.

### Refinement

C-bound H atoms were positioned geometrically (C—H = 0.95 Å) and refined as riding, with *U*_iso_(H) = 1.2*U*_eq_(C). The O-bound H atom was found in a difference Fourier map and refined freely. The refined O—H distance is 0.88 (2) Å.

### Crystal data

**Table:**

2C_7_H_4_ClNO_4_·C_4_H_4_N_2_	*F*(000) = 492.00
*M**_r_* = 483.22	*D*_x_ = 1.643 Mg m^−^^3^
Monoclinic, *P*2_1_/*c*	Mo *K*α radiation, λ = 0.71075 Å
Hall symbol: -P 2ybc	Cell parameters from 16875 reflections
*a* = 4.87662 (13) Å	θ = 3.0–30.1°
*b* = 13.5385 (3) Å	µ = 0.39 mm^−^^1^
*c* = 14.7981 (6) Å	*T* = 110 K
β = 90.858 (2)°	Block, colorless
*V* = 976.89 (5) Å^3^	0.35 × 0.15 × 0.11 mm
*Z* = 2	

### Data collection

**Table:**

Rigaku R-AXIS RAPID II diffractometer	2535 reflections with *I* > 2σ(*I*)
Detector resolution: 10.00 pixels mm^-1^	*R*_int_ = 0.032
ω scans	θ_max_ = 30.0°
Absorption correction: numerical (*NUMABS*; Higashi, 1999)	*h* = −6→6
*T*_min_ = 0.904, *T*_max_ = 0.958	*k* = −19→19
19734 measured reflections	*l* = −20→20
2833 independent reflections	

### Refinement

**Table:**

Refinement on *F*^2^	Primary atom site location: structure-invariant direct methods
Least-squares matrix: full	Secondary atom site location: difference Fourier map
*R*[*F*^2^ > 2σ(*F*^2^)] = 0.029	Hydrogen site location: inferred from neighbouring sites
*wR*(*F*^2^) = 0.081	H atoms treated by a mixture of independent and constrained refinement
*S* = 1.07	*w* = 1/[σ^2^(*F*_o_^2^) + (0.0499*P*)^2^ + 0.2334*P*] where *P* = (*F*_o_^2^ + 2*F*_c_^2^)/3
2833 reflections	(Δ/σ)_max_ = 0.001
149 parameters	Δρ_max_ = 0.51 e Å^−^^3^
0 restraints	Δρ_min_ = −0.39 e Å^−^^3^

### Special details

**Table:**

Geometry. All e.s.d.'s (except the e.s.d. in the dihedral angle between two l.s. planes) are estimated using the full covariance matrix. The cell e.s.d.'s are taken into account individually in the estimation of e.s.d.'s in distances, angles and torsion angles; correlations between e.s.d.'s in cell parameters are only used when they are defined by crystal symmetry. An approximate (isotropic) treatment of cell e.s.d.'s is used for estimating e.s.d.'s involving l.s. planes.
Refinement. Refinement of *F*^2^ against ALL reflections. The weighted *R*-factor *wR* and goodness of fit *S* are based on *F*^2^, conventional *R*-factors *R* are based on *F*, with *F* set to zero for negative *F*^2^. The threshold expression of *F*^2^ > σ(*F*^2^) is used only for calculating *R*-factors(gt) *etc*. and is not relevant to the choice of reflections for refinement. *R*-factors based on *F*^2^ are statistically about twice as large as those based on *F*, and *R*- factors based on ALL data will be even larger.

### Fractional atomic coordinates and isotropic or equivalent isotropic displacement parameters (Å^2^)

**Table:**

	*x*	*y*	*z*	*U*_iso_*/*U*_eq_	
Cl1	1.52398 (5)	0.692432 (19)	0.490381 (17)	0.02257 (8)	
O1	0.69354 (15)	0.47150 (5)	0.18863 (5)	0.02033 (15)	
O2	0.54429 (14)	0.62747 (5)	0.17436 (5)	0.01875 (15)	
O3	0.99168 (16)	0.77178 (6)	0.14572 (5)	0.02218 (16)	
O4	0.72288 (16)	0.82806 (6)	0.25018 (5)	0.02337 (17)	
N1	0.89159 (16)	0.76980 (6)	0.22167 (5)	0.01549 (16)	
N2	0.18864 (16)	0.54745 (6)	0.05617 (5)	0.01577 (16)	
C1	0.90441 (18)	0.59589 (7)	0.27806 (6)	0.01446 (17)	
C2	0.99232 (19)	0.69339 (6)	0.28474 (6)	0.01377 (17)	
C3	1.18351 (19)	0.72540 (7)	0.34825 (6)	0.01557 (18)	
H3	1.2416	0.7923	0.3504	0.019*	
C4	1.28706 (19)	0.65599 (7)	0.40871 (7)	0.01698 (18)	
C5	1.2038 (2)	0.55818 (7)	0.40559 (7)	0.01984 (19)	
H5	1.2757	0.5117	0.4478	0.024*	
C6	1.0143 (2)	0.52883 (7)	0.34015 (7)	0.01845 (19)	
H6	0.9584	0.4617	0.3376	0.022*	
C7	0.70336 (18)	0.55797 (7)	0.20900 (6)	0.01475 (17)	
C8	0.18281 (19)	0.44916 (7)	0.04969 (6)	0.01661 (18)	
H8	0.3099	0.4109	0.0841	0.020*	
C9	0.00621 (19)	0.59808 (7)	0.00657 (6)	0.01588 (18)	
H9	0.0056	0.6682	0.0098	0.019*	
H2	0.429 (4)	0.6024 (14)	0.1346 (14)	0.050 (5)*	

### Atomic displacement parameters (Å^2^)

**Table:**

	*U*^11^	*U*^22^	*U*^33^	*U*^12^	*U*^13^	*U*^23^
Cl1	0.02064 (13)	0.02541 (14)	0.02136 (13)	−0.00126 (9)	−0.00925 (9)	−0.00274 (9)
O1	0.0212 (3)	0.0153 (3)	0.0243 (4)	−0.0027 (3)	−0.0041 (3)	−0.0039 (3)
O2	0.0174 (3)	0.0171 (3)	0.0215 (4)	0.0001 (3)	−0.0072 (3)	−0.0038 (3)
O3	0.0290 (4)	0.0224 (4)	0.0151 (3)	−0.0004 (3)	0.0005 (3)	0.0007 (3)
O4	0.0249 (4)	0.0182 (3)	0.0271 (4)	0.0068 (3)	0.0004 (3)	−0.0010 (3)
N1	0.0167 (4)	0.0131 (3)	0.0166 (4)	−0.0019 (3)	−0.0031 (3)	−0.0010 (3)
N2	0.0148 (3)	0.0181 (4)	0.0144 (4)	−0.0023 (3)	−0.0012 (3)	−0.0013 (3)
C1	0.0139 (4)	0.0135 (4)	0.0160 (4)	−0.0009 (3)	−0.0011 (3)	−0.0025 (3)
C2	0.0138 (4)	0.0135 (4)	0.0139 (4)	0.0011 (3)	−0.0007 (3)	−0.0008 (3)
C3	0.0150 (4)	0.0150 (4)	0.0167 (4)	−0.0015 (3)	−0.0012 (3)	−0.0025 (3)
C4	0.0146 (4)	0.0199 (4)	0.0163 (4)	0.0005 (3)	−0.0037 (3)	−0.0024 (3)
C5	0.0207 (4)	0.0179 (4)	0.0208 (4)	0.0015 (4)	−0.0059 (4)	0.0014 (4)
C6	0.0200 (4)	0.0138 (4)	0.0215 (4)	−0.0005 (3)	−0.0036 (3)	−0.0002 (3)
C7	0.0131 (4)	0.0160 (4)	0.0151 (4)	−0.0022 (3)	0.0002 (3)	−0.0017 (3)
C8	0.0160 (4)	0.0178 (4)	0.0159 (4)	−0.0004 (3)	−0.0019 (3)	−0.0006 (3)
C9	0.0165 (4)	0.0154 (4)	0.0157 (4)	−0.0017 (3)	−0.0003 (3)	−0.0010 (3)

### Geometric parameters (Å, °)

**Table:**

Cl1—C4	1.7313 (10)	C2—C3	1.3836 (12)
O1—C7	1.2095 (11)	C3—C4	1.3875 (13)
O2—C7	1.3183 (11)	C3—H3	0.9500
O2—H2	0.88 (2)	C4—C5	1.3855 (14)
O3—N1	1.2323 (11)	C5—C6	1.3866 (13)
O4—N1	1.2195 (11)	C5—H5	0.9500
N1—C2	1.4727 (12)	C6—H6	0.9500
N2—C9	1.3343 (12)	C8—C9^i^	1.3888 (13)
N2—C8	1.3344 (13)	C8—H8	0.9500
C1—C2	1.3909 (12)	C9—C8^i^	1.3888 (13)
C1—C6	1.3932 (13)	C9—H9	0.9500
C1—C7	1.4964 (12)		
			
C7—O2—H2	110.7 (12)	C3—C4—Cl1	119.32 (7)
O4—N1—O3	125.45 (9)	C4—C5—C6	119.28 (9)
O4—N1—C2	117.09 (8)	C4—C5—H5	120.4
O3—N1—C2	117.41 (8)	C6—C5—H5	120.4
C9—N2—C8	117.35 (8)	C5—C6—C1	121.31 (9)
C2—C1—C6	117.15 (8)	C5—C6—H6	119.3
C2—C1—C7	124.91 (8)	C1—C6—H6	119.3
C6—C1—C7	117.93 (8)	O1—C7—O2	124.96 (9)
C3—C2—C1	123.33 (8)	O1—C7—C1	121.69 (9)
C3—C2—N1	115.18 (8)	O2—C7—C1	113.35 (8)
C1—C2—N1	121.47 (8)	N2—C8—C9^i^	121.03 (8)
C2—C3—C4	117.45 (9)	N2—C8—H8	119.5
C2—C3—H3	121.3	C9^i^—C8—H8	119.5
C4—C3—H3	121.3	N2—C9—C8^i^	121.62 (9)
C5—C4—C3	121.48 (9)	N2—C9—H9	119.2
C5—C4—Cl1	119.20 (7)	C8^i^—C9—H9	119.2
			
C6—C1—C2—C3	1.03 (14)	C3—C4—C5—C6	0.51 (15)
C7—C1—C2—C3	−178.46 (9)	Cl1—C4—C5—C6	−179.92 (8)
C6—C1—C2—N1	179.18 (9)	C4—C5—C6—C1	−0.57 (15)
C7—C1—C2—N1	−0.31 (14)	C2—C1—C6—C5	−0.16 (15)
O4—N1—C2—C3	−77.08 (11)	C7—C1—C6—C5	179.36 (9)
O3—N1—C2—C3	100.39 (10)	C2—C1—C7—O1	157.90 (10)
O4—N1—C2—C1	104.62 (10)	C6—C1—C7—O1	−21.58 (14)
O3—N1—C2—C1	−77.91 (11)	C2—C1—C7—O2	−22.11 (13)
C1—C2—C3—C4	−1.09 (14)	C6—C1—C7—O2	158.41 (9)
N1—C2—C3—C4	−179.35 (8)	C9—N2—C8—C9^i^	−0.01 (15)
C2—C3—C4—C5	0.29 (15)	C8—N2—C9—C8^i^	0.01 (15)
C2—C3—C4—Cl1	−179.28 (7)		

Symmetry codes: (i) −*x*, −*y*+1, −*z*.

### Hydrogen-bond geometry (Å, °)

**Table:**

*D*—H···*A*	*D*—H	H···*A*	*D*···*A*	*D*—H···*A*
O2—H2···N2	0.88 (2)	1.80 (2)	2.6739 (10)	178 (2)
C3—H3···O1^ii^	0.95	2.51	3.4305 (12)	162
C6—H6···O3^iii^	0.95	2.59	3.4865 (13)	157
C8—H8···O1	0.95	2.55	3.2201 (12)	128
C9—H9···O3^iv^	0.95	2.45	3.1273 (12)	128

Symmetry codes: (ii) −*x*+2, *y*+1/2, −*z*+1/2; (iii) −*x*+2, *y*−1/2, −*z*+1/2; (iv) *x*−1, *y*, *z*.
